# Evidence of Continued CD4+ and CD8+ T Cell Activity After SARS-COV-2 Clearance in a Late COVID-19 Pneumonia Heart Transplant Patient

**DOI:** 10.7759/cureus.24852

**Published:** 2022-05-09

**Authors:** Francisco R Klein, María F Renedo, Carlos A Vigliano

**Affiliations:** 1 Intensive Care Unit, Hospital Universitario Fundación Favaloro, Buenos Aires, ARG; 2 Intrathoracic Transplant, Hospital Universitario Fundación Favaloro, Buenos Aires, ARG; 3 Pathology, Hospital Universitario Fundación Favaloro, Buenos Aires, ARG

**Keywords:** covid-19, immuno suppresion, cd8 cells, cd4 t-cells, pathology, lung biopsy, heart transplantation, sars-cov-2

## Abstract

We have studied an unvaccinated heart transplant 64-year-old patient admitted for low-grade fever, dry cough, general malaise, and bilateral interstitial infiltrates, after two months of a diagnosis of coronavirus disease 2019 (COVID-19) bilateral pneumonia. A bronchoalveolar lavage and transbronchial biopsy were performed. Bacterial, mycotic and viral infections were ruled out including repeated reverse transcription polymerase chain reaction (RT-PCR) for severe acute respiratory syndrome coronavirus 2 (SARS-CoV-2).

Diffuse thickening of alveolar septa with fibrosis and infiltration of lymphocytes and macrophages into the alveolar septa with aggregates of CD4^+^ and CD8^+^ T cells with positive immunolabelling for granzyme B were observed, indicating a continuing cytotoxic process that might have induced proliferation and fibrosis.

An intense ongoing immunopathological cellular reaction, potentially triggered by SARS-CoV-2 overcoming the anti-inflammatory and immunomodulatory effects of the immunosuppressive drugs is suggested by these findings, opening to debate the usual approach of minimizing immunosuppression after COVID-19 in transplant patients when presence of SARS-CoV-2 has been ruled out.

## Introduction

The classic patterns of the pulmonary involvement induced by the coronavirus disease 2019 (COVID-19) in immunocompetent patients are bilateral pneumonia, diffuse alveolar damage and an eventual progression toward a fibroproliferative stage [1].

Most histopathological documentation has originated from autopsy reports [2], and recently, explanted lungs of lung transplant patients [3]. In a recent report, we have been able to show the histopathological evolution through sequential in-vivo transbronchial biopsies in an immunocompetent patient [4].

The mortality of heart transplant patients with COVID-19 has been reported to be between 15-33%, with in-hospital mortality reaching 41% [5,6], which is much higher for those with pulmonary complications, and respiratory failure being the leading cause of death [7]. Mortality decreases to 7.7% among recipients of two doses either of BNT162b2 (Pfizer) or Oxford/AstraZeneca (ChAdOx1-S) [8].

Nevertheless, until now, there has been limited information concerning the management of heart transplant patients with COVID-19 and no available in-vivo lung histopathological data.

## Case presentation

We recently studied a non-vaccinated 64-year-old male patient who had received a heart transplant in 2008 and was admitted in late December 2020 with a diagnosis of COVID-19 bilateral pneumonia without systemic involvement. Chest radiography on admission showed bilateral infiltrates. At that point, maintenance anti-rejection immunosuppression was achieved through tacrolimus 2 mg bi-daily plus mycophenolic acid 1080 mg bi-daily. Supplemental oxygen (6 L/min) was required to achieve a 94% oxygen saturation (SaO2). According to the RECOVERY trial findings [9], dexamethasone 6 mg/day for 10 days was indicated, followed by methylprednisolone 20 mg/day in addition to tacrolimus 1 mg bi-daily, while empirical antibiotic therapy was initiated. Blood, urine and sputum samples were negative for bacterial, mycotic and viral infections.

**Figure 1:**
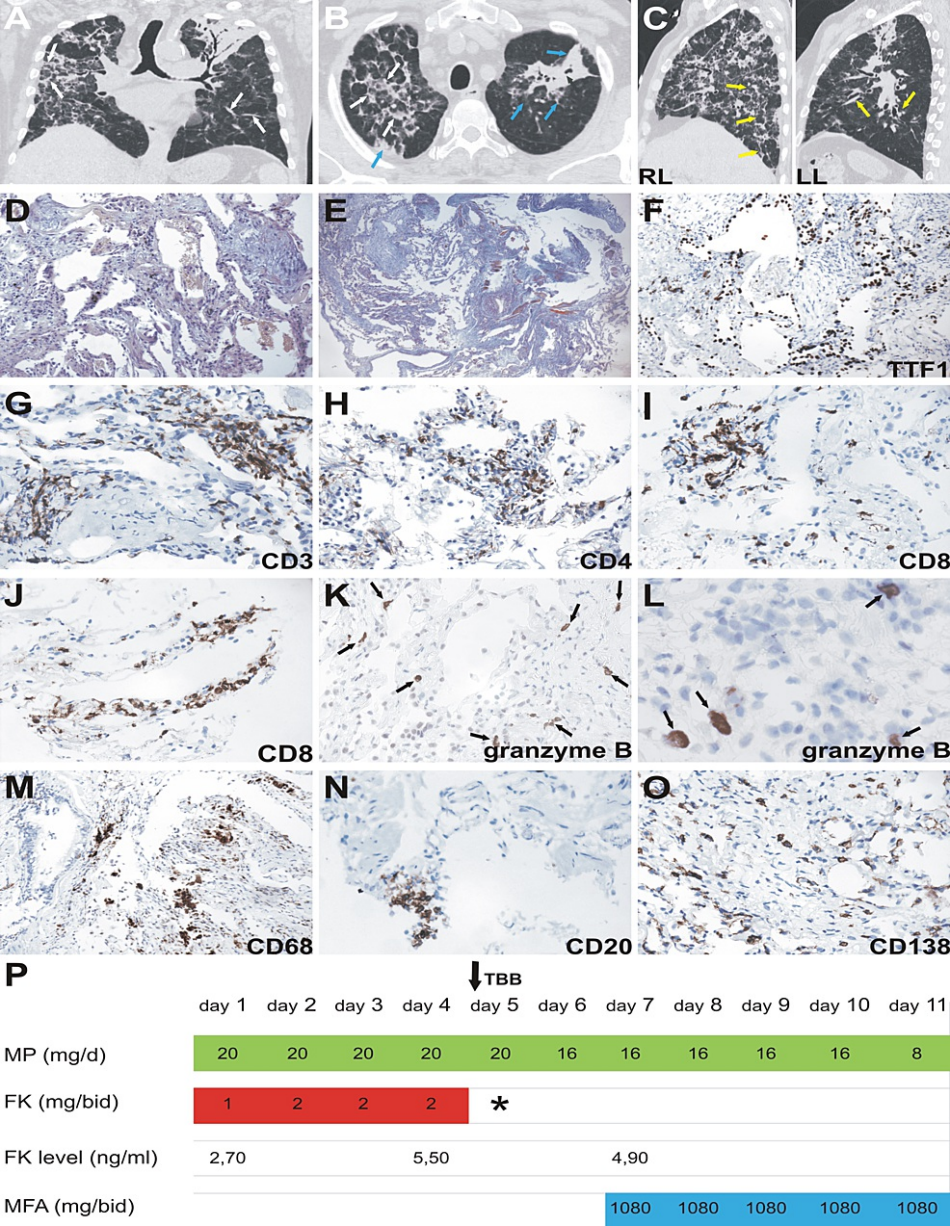
Tomographic, histological, immunophenotypic findings and immunosuppression regimen during hospitalization Chest CT scan. Hospital day 58.  A) Coronal view. Diffuse interstitial involvement with marked thickening of the interlobular septa (white arrows). B) Axial view. Diffuse interstitial involvement with marked thickening of the interlobular septa (white arrows) and alveolar consolidation area (green arrow) with bilateral ground glass areas of the upper left lobe (blue arrows). C) Lateral view. RL: right lung; LL: left lung. Traction bronchiectasis (yellow arrows). (D) Diffuse thickening of the alveolar septa with fibromyxoid material and mononuclear inflammatory infiltrate, (H&E x200). (E) Diffuse thickening of the alveolar septa with fibrosis, (Masson trichrome x100). (F) Type II pneumocyte hyperplasia, positive for TTF-1, (IHC x200). (G) Infiltrate of CD3+ T cells, in lung parenchima, (IHC x400). (H) Infiltrate of CD4+ T cells in alveolar septa, (IHC x400). (I, J) Infiltrate of CD8+ T cells in clusters and along the alveolar septa, (IHC x400). (K, L) CD8-positive cytotoxic cells (granzyme B+), (IHC x400). (M) Infiltrate of CD68+ cells (macrophages) in bronchial wall and lung parenchyma, (IHC x200). (N) Focal infiltrate of CD20+ B cells in alveolar septa, (IHC x400). (O) Lung parenchyma infiltrated with plasma cells, positive for CD138, (IHC x400). (P) In-hospital immunosuppressive scheme (days 58 to 68).  MP: methylprednisolone; FK: tacrolimus; MFA: mycophenolic acid; (*) Tacrolimus (FK 506) was temporarily discontinued due to kidney failure. TBB: transbronchial biopsy. IHC: Immunohistochemistry

Fifty-eight days after his initial admission, the patient was transferred to our centre. At admission, he presented with low-grade fever, dry cough and general malaise. On physical examination, the patient was thirsty, with a dry mouth, a temperature of 37.8 °C, a heart rate of 72 beats per minute and blood pressure of 125/70 mmHg. A body mass index of 21 kg/m2 was calculated, and the patient showed 93% SaO2 without supplemental oxygen (SaFiO2 of 443). Diffuse and bilateral Velcro-type crackles were detected by auscultation. No abdominal pathological findings or evidence of heart failure were observed. Bilateral pulmonary infiltrates were evident on chest radiography and computed tomography (CT) (Figure 1A-1C).

Because of the uncertain aetiology of pulmonary infiltrates and the possibility of opportunistic infections in a transplant patient, bronchoalveolar lavage and transbronchial biopsy were performed. Negative bacterial, mycotic and viral results (including repeated reverse-transcription polymerase chain reaction for severe acute respiratory syndrome coronavirus 2 (SARS-CoV-2) in both nasopharyngeal swabs and bronchoalveolar lavage samples) were obtained.

Pathologically, as shown in Figure 1D-1F, lung biopsies showed diffuse thickening of the alveolar septa with fibromyxoid material, type II pneumocyte hyperplasia positive for thyroid transcription factor-1 (TTF-1) and mononuclear inflammatory infiltrates. The infiltrate was predominantly composed of CD3+ T cells (Figure 1G) and macrophages. The persistent cellular immunity activity was consistent with T cell aggregates composed of abundant CD4+ (Figure 1H) and CD8+ T cells. CD8+ T cells were predominantly distributed along the alveolar septa (Figure 1I, 1J), with positive granzyme B immunohistochemistry (Figure 1K, 1L). The infiltration of CD68+ cells into the bronchial wall, alveolar septa and alveoli was noteworthy (Figure 1M). The CD20+ B cells were arranged into a few small clusters, while scattered plasma cells positive for CD138 infiltrated the lung parenchyma (Figure 1N, 1O). Active proliferative and fibrotic processes were histologically demonstrated by the expansion of fibroblasts, with extensive interstitial collagen deposition. There was no evidence of microthrombi within the pulmonary vessels by direct observation of fibrin staining or immunohistochemical staining for antibodies against platelet glycoprotein III a (CD61). No findings were compatible with overt endotheliitis. Immunohistochemical analysis of caspase-3 showed negative results. The search for complement component 4d (C4d) deposition in microvessels using anti-C4d antibodies yielded negative results.

The patient showed progressive improvement of his clinical status being discharged after 10 days with a SaFiO2 of 452.4 breathing room air with the administration of mycophenolic acid 1080 mg/day and prednisone 8 mg/day (Figure 1P).

## Discussion

The pulmonary inflammatory, organising and fibroproliferative consequences of COVID-19 in immunocompetent patients have been described mainly from autopsy studies with only a few in-vivo published data [10,11]. Even less information is available on lung histopathological and immune responses in immunosuppressed patients.

Patients undergoing transplantation require immunosuppression to avoid donor organ rejection. Immunosuppressants affect viral replication, as well as inflammatory and fibro-proliferative tissue responses. Thus, decision-making regarding the adjustment of immunosuppression in transplant patients with COVID-19 remains open to debate [12].

The most frequently described clinical patterns in immunocompetent deceased patients with COVID-19 have been bilateral pneumonia, diffuse alveolar damage and a late fibroproliferative stage [13]. A subgroup with bilateral pneumonia and diffuse alveolar damage shows a high mortality rate, comprising patients with a high viral load and high cytokine expression, intense multi-system involvement and early-onset diffuse parenchymal damage. In the second subgroup, patients with a fibroproliferative response present with a lower viral load and cytokine expression and a protracted disease evolution [14].

In our case, post-COVID-19 pulmonary fibrosis was diagnosed according to the criteria suggested by Huang et al. (extensive and persistent fibrotic changes, including parenchymal bands, irregular interfaces, reticular opacities, and traction bronchiectasis with honeycombing images) [15].

It has been shown that in COVID-19 pneumonia, an initial acute viral response phase is activated, giving rise to diffuse alveolar damage, followed by disease progression modulated by the host inflammatory response [13]. A late phase may result in an overactive immune system response driven by T cells and macrophage activation, which could trigger the organising and proliferative lung changes that are characteristic of this state [16,17]. Our findings of a positive granzyme B immunolabelling of CD8+ lymphocytes indicate a continued cytotoxic activity potentially triggering the progressive proliferative and fibrotic process.

Histologically, COVID-19 pulmonary involvement has been divided into three principal patterns: a) predominantly epithelial, showing epithelial cell death with secondary hyperplasic changes and diffuse alveolar damage; b) predominantly vascular, showing microvascular damage, micro-thrombi and acute fibrinous and organising pneumonia; and c) predominantly fibrotic, with evidence of interstitial fibrosis and alveolar obliteration. The pathogenesis of the fibrotic changes involves repetitive damage to the alveolar epithelium leading to an ineffective repair process with epithelial dysfunction [18]. Fibrotic changes usually appear at a late stage, generally three weeks after the initial respiratory symptoms. However, most lung samples may show elements of these different categories and stages in variable proportions according to disease evolution [13].

Data from a recently published review show that approximately 20% of COVID-19 patients progress to fibrotic sequelae that persist until the one-year follow-up [19].

Heart transplant patients represent a unique population of immunosuppressed subjects in which SARS-CoV-2 may cause an unpredictable clinical course of infection. Histopathological data are still lacking for vulnerable patients [7].

In this late in-vivo transbronchial biopsy obtained from a COVID-19 pneumonia immunosuppressed heart transplant patient, we were able to demonstrate extensive proliferative and fibrotic changes in the absence of evidence of viral presence two months after the initial viral infection.

Even after viral clearance, our findings show persistent cellular immune response and ongoing pro-organising and pro-fibrotic processes, which might have been triggered by the virus and not halted by patient-adjusted immunosuppression. The cellular inflammatory reaction seems particularly noteworthy based on the abundance of CD8+ T cells with evident granzyme B immunohistochemical staining. This finding is particularly interesting considering its development despite the patient receiving immunosuppressive drugs (steroids, mycophenolate and tacrolimus) that interfere with T- and B-lymphocyte activity and possess antiproliferative actions.

It is not easy to describe major histopathological or imaging differences between the fibroproliferative phases of COVID-19 pneumonia and the late stages of idiopathic interstitial pneumonias which should always bring up the issue of its nosologic characterization and eventually the use of a common therapeutical approach like steroids or anti-fibrotic agents [20].

In transplant patients, reduction of immunosuppressive medication during the initial stages of the disease appears to be a reasonable approach to control acute viral replication and could influence its clinical expression. On the other hand, since much of the lung damage observed in later stages could result from immune hyperactivation driven by T cells, a sharp decrease in immunosuppression when no active viral replication is demonstrable might contribute to an increased organising and/or fibroproliferative delayed response [16].

## Conclusions

The decisions pertaining to the continuation, modification or discontinuation of immunosuppressants in transplant patients with COVID-19 remain controversial and still lack sound scientific evidence. Our study highlights a persistent cellular inflammatory response based on the abundance of CD4+ and CD8+ T cells with evident granzyme B immunohistochemical staining in a late COVID-19 pneumonia heart transplant patient.

These findings suggest that the ongoing immunopathological cellular and humoral reactions triggered by SARS-CoV-2 are resistant to the anti-inflammatory and immunomodulatory effects of immunosuppressive drugs and could contribute to an increase in lung proliferative and fibrotic changes in the absence of evidence of viral presence.
